# Supplementary material for: Host oxidative stress primes mycobacteria for rapid antibiotic resistance evolution
Source: Nat Commun. 2026 May 7;17:4106. doi: 10.1038/s41467-026-72496-4 (PMC13153227; doi:10.1038/s41467-026-72496-4)
Supplement: Supplementary file 1 — Supplementary Information [file 41467_2026_72496_MOESM1_ESM.pdf]

Supplementary Information

Host oxidative stress primes mycobacteria for rapid antibiotic resistance evolution

Evan Pepper-Tunick[1,2], Vivek Srinivas[1], Fred D. Mast[3,4], Song Li[3], Sagan Russ[1], Weston Hanson[1], Amy D. Zamora[1], Wei-Ju Wu[1], Matthew Silcocks[5], Dang Thi Minh Ha[6], Sarah J. Dunstan[5], Thuong Nguyen Thuy Thuong[7,8], Serdar Turkarslan[1], John D. Aitchison[3,4,9], Mario L. Arrieta-Ortiz[1]*, Nitin S. Baliga[1,2,10,11,12,13]*

[1]Institute for Systems Biology, Seattle, WA, USA

[2]Molecular Engineering and Sciences Institute, University of Washington, Seattle, WA, USA

[3]Seattle Children's Research Institute, Seattle, WA, USA

[4]Department of Pediatrics, University of Washington, Seattle, WA, USA

[5]Department of Infectious Diseases, University of Melbourne at the Peter Doherty Institute for Infection and Immunity, Parkville, Victoria, Australia

[6]Pham Ngoc Thach Hospital for TB and Lung Disease, District 5, Ho Chi Minh City, Vietnam

[7]Oxford University Clinical Research Unit, Hospital for Tropical Diseases, District 5, Ho Chi Minh City, Vietnam

[8]Nuffield Department of Medicine, Centre for Tropical Medicine and Global Health, University of Oxford, Oxford, United Kingdom

[9]Department of Biochemistry, University of Washington, Seattle, WA, USA

[10]Department of Biology, University of Washington, Seattle, WA, USA

[11]Department of Microbiology, University of Washington, Seattle, WA, USA

[12]Department of Civil & Environmental Engineering, University of Washington, Seattle, WA, USA

[13]Lawrence Berkeley National Laboratory, Berkeley, CA, USA

*Corresponding authors

Nitin. S. Baliga: nitin.baliga@isbscience.org

Mario L. Arrieta-Ortiz: mario.arrieta-ortiz@isbscience.org

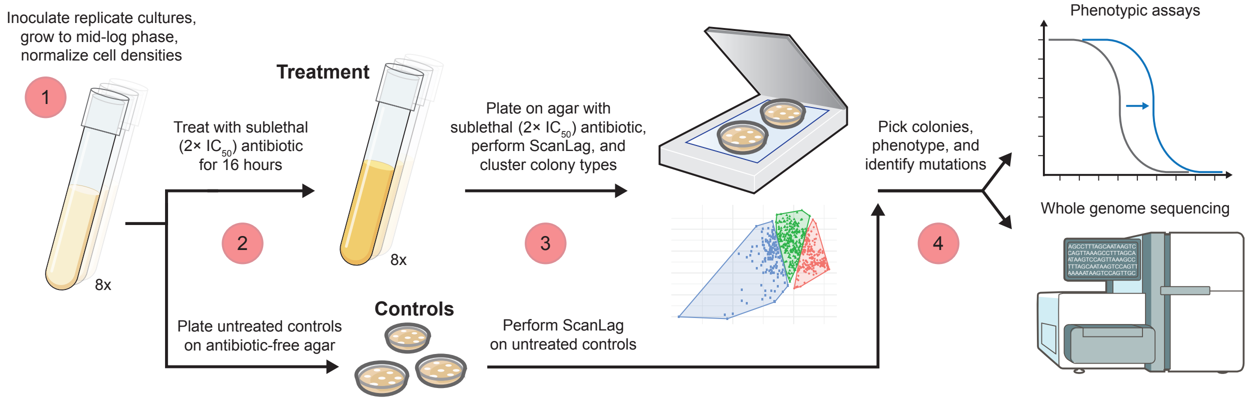

Supplementary Fig. 1: A sub-inhibitory antibiotic treatment to enrich for pre-resistant mutants. (1) Biological replicates of Msm mc^{2}155 cultures reach log phase (OD_{600} 0.6–1.0) and are then OD_{600} normalized into fresh liquid media. **(2)** Before adding antibiotic, untreated control aliquots of each culture are plated on antibiotic free agar. Then, 2× IC_{50} INH is added to all treated samples. Cultures are incubated at 37ºC for 16 hours **(3)** Treated cultures are plated out on solid 7H10 agar with 2× IC_{50} INH and ScanLag is performed alongside untreated controls on antibiotic free agar. ScanLag data is then processed and colonies from treated cultures are clustered based on their growth characteristics. **(4)** Representative colonies from each cluster are then picked, grown isogenically, phenotyped for shifts in their IC_{50}, and sequenced using whole genome sequencing technologies to identify mutation(s) that confer the corresponding phenotype. Illustrations of petri dish[1] and next gen sequencer[2] from NIAID NIH BioArt Source (bioart.niaid.nih.gov/bioart/404 and bioart.niaid.nih.gov/bioart/386, respectively). All other artwork in this figure was created by the authors.

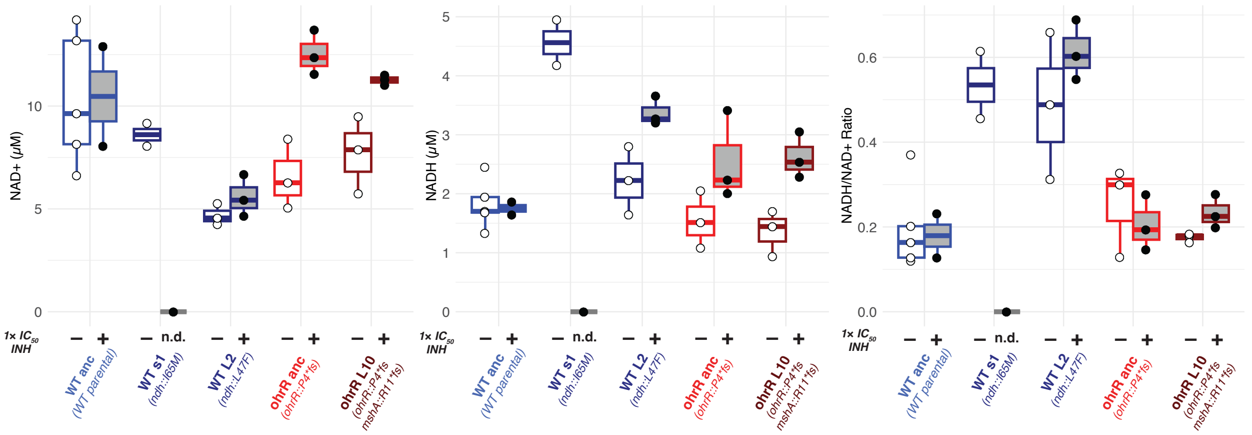

Supplementary Fig. 2: Redox chemistry is altered in various INH^R strains, depending on their genetic background. NAD+ quantification, NADH quantification, and NADH/NAD+ ratio during log phase growth of the wildtype, wildtype-derived INH^R strains s1 (*ndh::I65M*) and L2 (*ndh::L47F*), *ohrR::P4*fs* LLRT strain, and its derived INH^R strain L10 (*ohrR::P4*fs – mshA::R11*fs*) in the absence (indicated with "(–)") and presence (indicated with "(+)") of 1× IC_{50} INH. Due to sample loss, INH-treated WT anc and untreated WT s1 are represented by two biological replicates. Otherwise, all data are representative of n = 3 independent experiments. Strains are labeled with the name of the background from which they were derived and their genotype relative to the wildtype parental strain. Point mutants are named by their colony size (s for small, L for large) and the isolate number. Box plots indicate the median (center line), 25th and 75th percentiles (bounds of box), and whiskers extending to ±1.5× the IQR; overlayed by the measured value for each biological replicate (points). Source data are provided as a Source Data file.

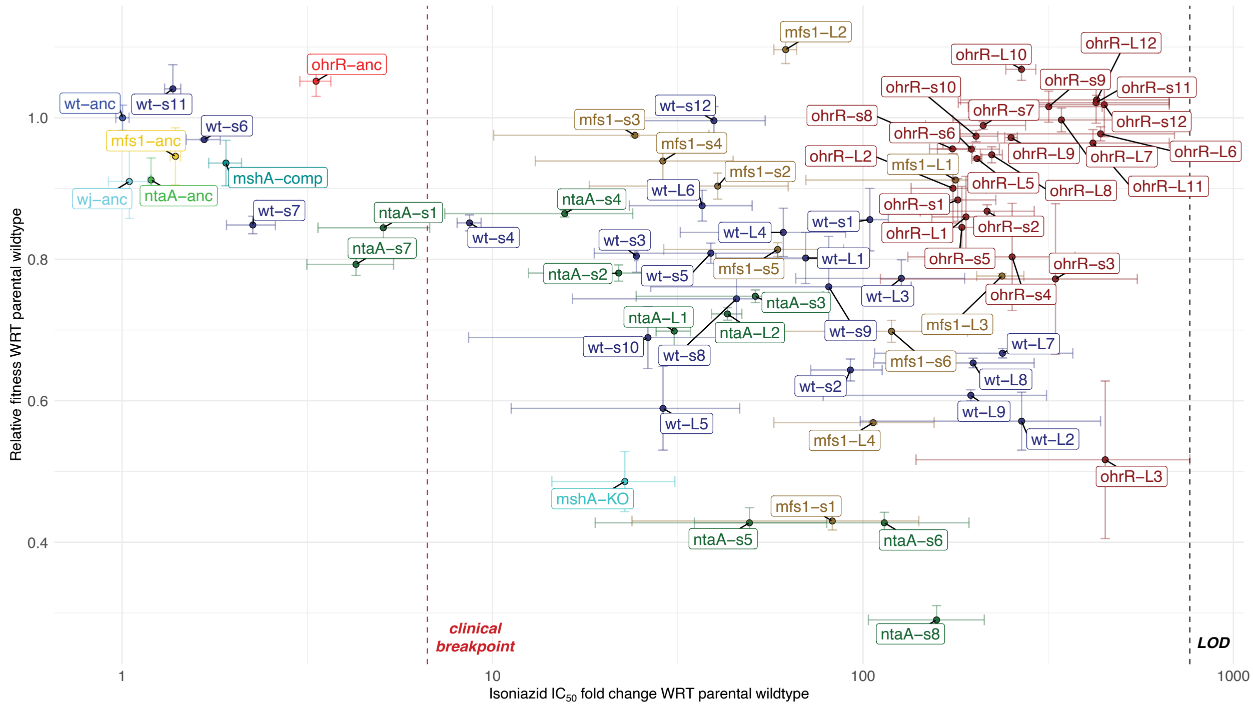

Supplementary Fig. 3: Scatter plot of relative fold changes in INH IC_{50} and fitness in the absence of antibiotic of all INH^R isolates derived from wildtype and LLRT backgrounds. Various strains shown were grown to log phase and phenotyped in a dose response assay to quantify the INH IC_{50} fold change and relative fitness with respect to the wildtype strain which they were originally derived from. Error bars represent the mean ± standard error across two replicates in two independent experiments (n = 4). The black horizontal dashed line in represents the upper limit of detection (LOD) for resistance. The red horizontal line represents the clinical breakpoint for high-level INH resistance (≥ 6.6× fold change in IC50). Strain labels: wt-anc: Nitin Baliga Lab wildtype Msm mc^{2}155; wj-anc: William Jacobs Lab wildtype Msm mc^{2}155; *mshA*-KO: Δ*mshA* knockout derived from W.J. wildtype; *mshA*-comp: Δ*mshA* pMV361::*mshA* complemented strain derived from W.J. wildtype; mfs1-anc: *mfs1::G105D*; ntaA-anc: *ntaA_5::E50*fs*; ohrR-anc: *ohrR::P4*fs*. All remaining strains were derived from fluctuation assay of wildtype or LLRT strains. Source data are provided as a Source Data file.

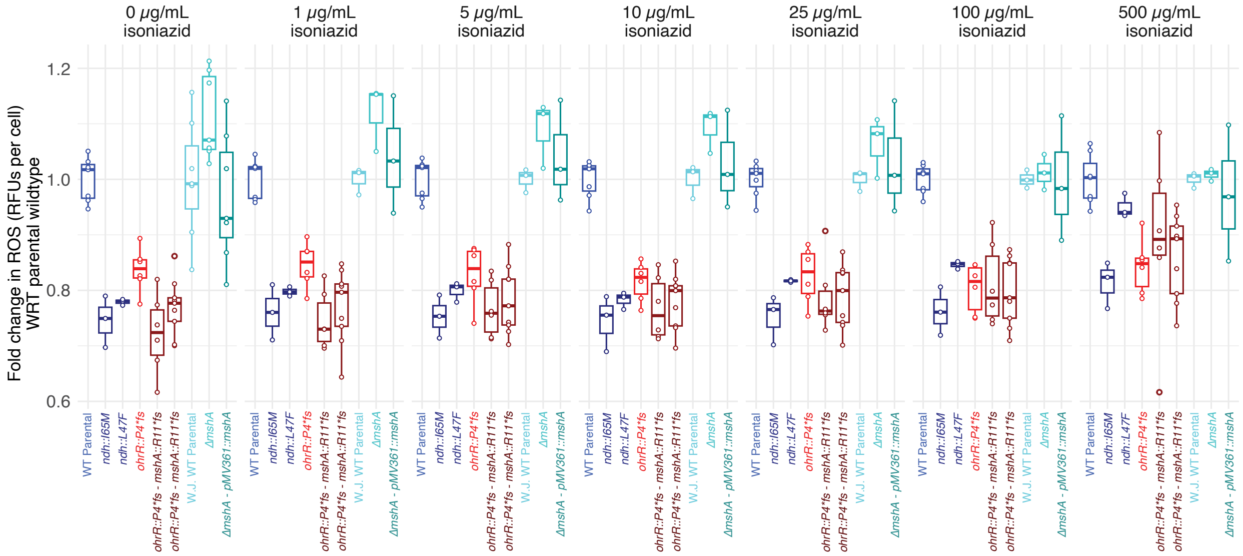

Supplementary Fig. 4: Endogenous ROS levels differ in INH^R strains relative to their susceptible ancestral background. Various strains shown were grown to log phase (OD_{600} 0.6–1.0) and then normalized to 8.0 x 10^6 cells / mL before exposing them to the fluorescent dye 2',7'-dichlorodihydrofluorescein diacetate (H2DCFDA), with or without INH at the specified concentration (0, 1, 5, 10, 25, 100, or 500 µg/mL). RFUs were measured after 1 hour of incubation at 37ºC and normalized back to the cell count. Fold change RFUs for each strain is the ratio of the RFUs for a given sample relative to the RFUs of the wildtype strain from which each strain was derived, under the same conditions. Box plots indicate the median (center line), 25th and 75th percentiles (bounds of box), and whiskers extending to ±1.5× the IQR; overlayed by the measured value for each biological replicate (points), across at least three biological replicates. Source data are provided as a Source Data file.

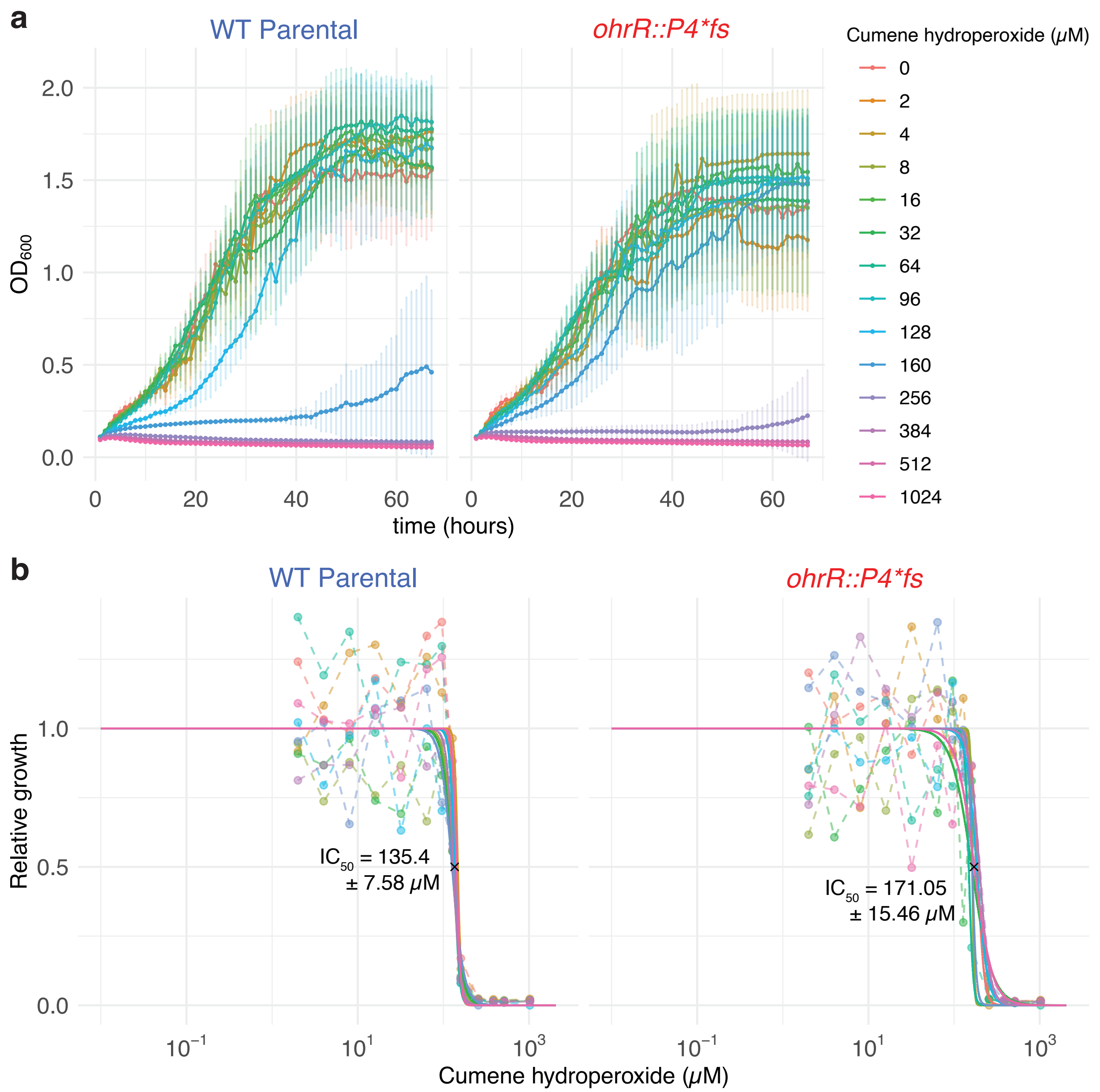

Supplementary Fig. 5: The *ohrR::P4*fs* mutant is low-level resistant to cumene hydroperoxide. Dose response assay of wildtype and *ohrR::P4*fs* in cumene hydroperoxide. Strains shown were grown to log phase (OD_{600} 0.6–1.0) and then normalized to OD_{600} 0.02 before subjecting to a dose escalation of cumene hydroperoxide. OD_{600} was measured over 48 hours of growth. Growth curves **(a)** and dose response curves **(b)** were generated as described in the Methods. Error bars in **(a)** represent the mean ± standard deviation across n = 3 independent experiments. The calculated IC_{50} in **(b)** the average across all replicates ± standard deviation. Source data are provided as a Source Data file.

Supplementary References

1. "NIAID Visual & Medical Arts. 08/28/2024. Petri Dish. NIAID BioArt Source. bioart.niaid.nih.gov/bioart/404"
2. "NIAID Visual & Medical Arts. 08/28/2024. Next Gen Sequencer. NIAID BioArt Source. bioart.niaid.nih.gov/bioart/386"
