# Supplementary material for: Host oxidative stress primes mycobacteria for rapid antibiotic resistance evolution
Source: Nat Commun. 2026 May 7;17:4106. doi: 10.1038/s41467-026-72496-4 (PMC13153227; doi:10.1038/s41467-026-72496-4)
Supplement: Supplementary file 2 — Description of Additional Supplementary File [file 41467_2026_72496_MOESM2_ESM.pdf]

Description of Additional Supplementary Files:

Supplementary Data 1 :

Locus: Mtb H37Rv gene or intergenic region involved in the OSR network with significant BP. BP: Bayes Probability. FDR p value: FDR-corrected p value of BP. Role in antibiotic resistance or the OSR: Summarized role in antibiotic resistance or the oxidative stress response network of the specific gene, intergenic region, or mutations within the locus. Supporting Literature: Existing literature, if any, supporting the mentioned role.

Supplementary Data 2 :

Legend CRISPRi treatment: CRISPRi library treatment (Li, S. et al. 2022) condition in which enrichment test was performed and genes were grouped. In Li, S. et al. 2022, the FDR-adjusted p values were previously reported by the authors. “Enriched” genes had FDR ≤ 0.01 and log2 fold change ≥ 1, “Depleted” genes had FDR ≤ 0.01 and log2 fold change ≤ -1, “Responder” genes were either enriched or depleted with FDR ≤ 0.01. CLR: clarithromycin; INH: isoniazid; STR: streptomycin; EMB: ethambutol; RIF: rifampicin; VAN: vancomycin; BDQ: bedaquiline; LZD: linezolid. BP vs. CRISPRi hypergeometric test p value: Significant one-sided hypergeometric test p value (p value ≤ 0.05) for overrepresentation of the 207 genes with significant Bayes Probability (BP) (FDR ≤ 0.05) among CRISPRi knockdown strains under various treatment conditions.
