# Supplementary material for: Host oxidative stress primes mycobacteria for rapid antibiotic resistance evolution
Source: Nat Commun. 2026 May 7;17:4106. doi: 10.1038/s41467-026-72496-4 (PMC13153227; doi:10.1038/s41467-026-72496-4)
Supplement: Supplementary file 5 — Reporting summary [file 41467_2026_72496_MOESM5_ESM.pdf]

Corresponding author(s): Nitin S. Baliga
Last updated by author(s): Mar 31, 2026

Reporting Summary

Nature Portfolio wishes to improve the reproducibility of the work that we publish. This form provides structure for consistency and transparency in reporting. For further information on Nature Portfolio policies, see our Editorial Policies and the Editorial Policy Checklist.

Statistics

For all statistical analyses, confirm that the following items are present in the figure legend, table legend, main text, or Methods section.

n/a	Confirmed	
☐	☒	The exact sample size (*n*) for each experimental group/condition, given as a discrete number and unit of measurement
☐	☒	A statement on whether measurements were taken from distinct samples or whether the same sample was measured repeatedly
☐	☒	The statistical test(s) used AND whether they are one- or two-sided<br>*Only common tests should be described solely by name; describe more complex techniques in the Methods section.*
☒	☐	A description of all covariates tested
☐	☒	A description of any assumptions or corrections, such as tests of normality and adjustment for multiple comparisons
☐	☒	A full description of the statistical parameters including central tendency (e.g. means) or other basic estimates (e.g. regression coefficient) AND variation (e.g. standard deviation) or associated estimates of uncertainty (e.g. confidence intervals)
☐	☒	For null hypothesis testing, the test statistic (e.g. *F*, *t*, *r*) with confidence intervals, effect sizes, degrees of freedom and *P* value noted<br>*Give P values as exact values whenever suitable.*
☒	☐	For Bayesian analysis, information on the choice of priors and Markov chain Monte Carlo settings
☐	☒	For hierarchical and complex designs, identification of the appropriate level for tests and full reporting of outcomes
☐	☒	Estimates of effect sizes (e.g. Cohen's *d*, Pearson's *r*), indicating how they were calculated

Our web collection on statistics for biologists contains articles on many of the points above.

Software and code

Policy information about availability of computer code

Data collection	Data collection was done with custom Python, R, and shell scripts dependent on third-party tools.
Data analysis	- Microsoft Excel (365)<br>- Google Collab<br>- Python (version 3.12.12)<br>- cv2 (version 4.12.0)<br>- numpy (version 2.0.2)<br>- imutils (version 0.5.4)<br>- skimage (version 0.25.2)<br>- pandas (version 2.2.2)<br>- matplotlib (version 3.10.0)<br>- seaborn (version 0.13.2)<br>- SciPy (version 1.16.3)<br>- ScanLag (https://github.com/baliga-lab/Scanlag)<br>- R (version 4.1.1)<br>- scales (version 1.4.0)<br>- ggpubr (version 0.6.1)<br>- rstatix (version 0.7.2)<br>- naniar (version 1.1.0)<br>- factoextra (version 1.0.7)<br>- knitr (version 1.50)

- drc (version 3.0-1)
- lubridate (version 1.9.4)
- forcats (version 1.0.0)
- stringr (version 1.5.1)
- dplyr (version 1.1.4)
- purrr (version 1.1.0)
- readr (version 2.1.5)
- tidyr (version 1.3.1)
- tibble (version 3.3.0)
- tidyverse (version 2.0.0)
- growthcurver (version 0.3.1)
- ggridges (version 0.5.7)
- ggplot2 (version 3.5.2)
- ggrepel (version 0.9.6)
- ggupset (version 0.4.1)
- see (version 0.11.0)
- viridis (version 0.6.5)
- seqinr (version 4.2-36)
- Biostrings (version 2.72.1)
- FALCOR (https://lianglab.brocku.ca/FALCOR/)
- Custom variant calling pipeline (https://github.com/baliga-lab/bwa_pipeline)
- TrimGalore (version 0.6.10)
- BWA (version 0.7.17-r1188)
- Picard (version 2.27.5)
- GATK (version 4.3.0.0)
- Varscan (version 2.4.3)
- Samtools (version 1.6)
- SnpEff (version 5.1d)
- SRA toolkit, fasterq-dump (version 3.2.1)
- TB-Profiler (version 6.3.0)
- vcfpy (version 0.13.8)
- mmseqs (version 14.7e284)
- perl (version 5.32.1)
- Cytoscape (version 3.10.3)

For manuscripts utilizing custom algorithms or software that are central to the research but not yet described in published literature, software must be made available to editors and reviewers. We strongly encourage code deposition in a community repository (e.g. GitHub). See the Nature Portfolio guidelines for submitting code & software for further information.

Data

Policy information about availability of data

All manuscripts must include a data availability statement. This statement should provide the following information, where applicable:

- Accession codes, unique identifiers, or web links for publicly available datasets
- A description of any restrictions on data availability
- For clinical datasets or third party data, please ensure that the statement adheres to our policy

All custom code, processed data, computational analyses, variant calls, and supplementary materials generated in this study are publicly available through a Zenodo archive of a GitHub release (v1.2) [https://doi.org/10.5281/zenodo.19355216]. All raw data underlying the figures are provided as a Source Data file with this paper. Raw whole-genome sequencing data for Mycobacterium smegmatis strains generated in this study have been deposited in the Sequence Read Archive (SRA) under BioProject accession PRJNA1363199 [https://www.ncbi.nlm.nih.gov/bioproject/PRJNA1363199]. All strains generated in this study are available for reuse and can be obtained from the corresponding authors upon request. Whole-genome sequencing data for previously published Mycobacterium tuberculosis isolates used in this study are available through the SRA under BioProject accessions PRJNA1028637 [https://www.ncbi.nlm.nih.gov/bioproject/PRJNA1028637] and PRJNA355614 [https://www.ncbi.nlm.nih.gov/bioproject/PRJNA355614]. Individual SRA accession numbers for these isolates are provided in the Source Data file. The Mycobacterium smegmatis reference genome (NC_008596.1) is available from NCBI [https://www.ncbi.nlm.nih.gov/nuccore/NC_008596.1]. The inferred Mycobacterium tuberculosis ancestral reference genome (Green et al., 2023) was used for alignment and variant calling. Large intermediate datasets (e.g., Bayesian null distributions) are available via Zenodo at [10.5281/zenodo.19207254].

Research involving human participants, their data, or biological material

Policy information about studies with human participants or human data. See also policy information about sex, gender (identity/presentation), and sexual orientation and race, ethnicity and racism.

Reporting on sex and gender	*Use the terms sex (biological attribute) and gender (shaped by social and cultural circumstances) carefully in order to avoid confusing both terms. Indicate if findings apply to only one sex or gender; describe whether sex and gender were considered in study design; whether sex and/or gender was determined based on self-reporting or assigned and methods used.*<br>*Provide in the source data disaggregated sex and gender data, where this information has been collected, and if consent has been obtained for sharing of individual-level data; provide overall numbers in this Reporting Summary. Please state if this information has not been collected.*<br>*Report sex- and gender-based analyses where performed, justify reasons for lack of sex- and gender-based analysis.*
Reporting on race, ethnicity, or other socially relevant	*Please specify the socially constructed or socially relevant categorization variable(s) used in your manuscript and explain why they were used. Please note that such variables should not be used as proxies for other socially constructed/relevant variables (for example, race or ethnicity should not be used as a proxy for socioeconomic status).*

groupings	*Provide clear definitions of the relevant terms used, how they were provided (by the participants/respondents, the researchers, or third parties), and the method(s) used to classify people into the different categories (e.g. self-report, census or administrative data, social media data, etc.)<br>Please provide details about how you controlled for confounding variables in your analyses.*
Population characteristics	*Describe the covariate-relevant population characteristics of the human research participants (e.g. age, genotypic information, past and current diagnosis and treatment categories). If you filled out the behavioural & social sciences study design questions and have nothing to add here, write "See above."*
Recruitment	*Describe how participants were recruited. Outline any potential self-selection bias or other biases that may be present and how these are likely to impact results.*
Ethics oversight	*Identify the organization(s) that approved the study protocol.*

Note that full information on the approval of the study protocol must also be provided in the manuscript.

Field-specific reporting

Please select the one below that is the best fit for your research. If you are not sure, read the appropriate sections before making your selection.

☒ Life sciences ☐ Behavioural & social sciences ☐ Ecological, evolutionary & environmental sciences

For a reference copy of the document with all sections, see nature.com/documents/nr-reporting-summary-flat.pdf

Life sciences study design

All studies must disclose on these points even when the disclosure is negative.

Sample size	No statistical method was used to predetermine sample size. Sample sizes were selected based on prior experience with similar experimental systems and standard practices in mycobacterial microbiology to ensure sufficient reproducibility and statistical power. Eight independent biological replicates with at least three technical replicates each were used in the initial one-step selection experiment. Due to the very large number of isolates screened, dose response assays for fitness and IC50 determination were performed with at least two technical replicates across two independent biological replicates (four replicate assessments in total). For tolerance assays, 12 biological replicates with at least two technical replicates each were used for CFU quantification. Fluctuation assays with and without the cumene hydroperoxide pretreatment were performed using 12 biological replicates inoculated at low cell density, with CFUs quantified across three technical replicates before and after selection with high-dose antibiotic. For NADH/NAD+ quantification, three biological replicates were assessed for each strain. For ROS measurements, at least three biological replicates per strain were assessed across two technical replicates. These sample sizes were sufficient to ensure reproducibility across independent experiments and to capture consistent phenotypic differences between strains and conditions. The final dose response assay in wildtype and ohrR::P4*fs in cumene hydroperoxide was performed with three technical replicates of three biological replicates.
Data exclusions	No data were excluded from the analyses except where samples were lost during experimental processing. Specifically, in Figure 4A and Supplementary Fig. 2, only two biological replicates were recovered for wildtype treated with 1× IC50 isoniazid and strain WT s1 untreated due to sample loss. Data from WT s1 and WT L2 untreated were aggregated due to their highly similar genomic backgrounds. WT s1 was not measured under treated conditions. All other NADH/NAD+ quantification experiments were performed with three biological replicates.
Replication	All experiments were independently replicated as described in the Methods and figure legends. Biological replicates represent independently grown cultures, and technical replicates represent repeated measurements within each biological replicate. Key findings were reproduced across independent experiments.
Randomization	The experiments were not randomized. For the computational analysis, statistical significance of Bayesian probabilities was assessed by comparison to a null distribution generated from > 5000 iterations of randomized mutation profiles across samples. Resulting p values were corrected for multiple hypothesis testing using false discovery rate (FDR) adjustment.
Blinding	The investigators were not blinded to allocation during experiments and outcome assessment. Measurements including optical density, colony-forming units, and fluorescence-based assays were quantitative and instrument-based, minimizing the potential for observer bias.

Reporting for specific materials, systems and methods

We require information from authors about some types of materials, experimental systems and methods used in many studies. Here, indicate whether each material, system or method listed is relevant to your study. If you are not sure if a list item applies to your research, read the appropriate section before selecting a response.

Materials & experimental systems

n/a	Involved in the study
☒	☐ Antibodies
☒	☐ Eukaryotic cell lines
☒	☐ Palaeontology and archaeology
☒	☐ Animals and other organisms
☒	☐ Clinical data
☒	☐ Dual use research of concern
☒	☐ Plants

Methods

n/a	Involved in the study
☒	☐ ChIP-seq
☒	☐ Flow cytometry
☒	☐ MRI-based neuroimaging

Plants

Seed stocks	*Report on the source of all seed stocks or other plant material used. If applicable, state the seed stock centre and catalogue number. If plant specimens were collected from the field, describe the collection location, date and sampling procedures.*
Novel plant genotypes	*Describe the methods by which all novel plant genotypes were produced. This includes those generated by transgenic approaches, gene editing, chemical/radiation-based mutagenesis and hybridization. For transgenic lines, describe the transformation method, the number of independent lines analyzed and the generation upon which experiments were performed. For gene-edited lines, describe the editor used, the endogenous sequence targeted for editing, the targeting guide RNA sequence (if applicable) and how the editor was applied.*
Authentication	*Describe any authentication procedures for each seed stock used or novel genotype generated. Describe any experiments used to assess the effect of a mutation and, where applicable, how potential secondary effects (e.g. second site T-DNA insertions, mosiacism, off-target gene editing) were examined.*
